# Supplementary material for: The interactome of intact mitochondria by cross-linking mass spectrometry provides evidence for coexisting respiratory supercomplexes
Source: Mol Cell Proteomics. 2017 Dec 8;17(2):216–32. doi: 10.1074/mcp.RA117.000470 (PMC5795388; doi:10.1074/mcp.RA117.000470)
Supplement: Supplemental Data [file supp_RA117.000470_133922_0_supp_23978_fzffwf.zip › spectra_annotation/mito_DR_spectra_annotation/27-1-6-1-8-1.pdf]

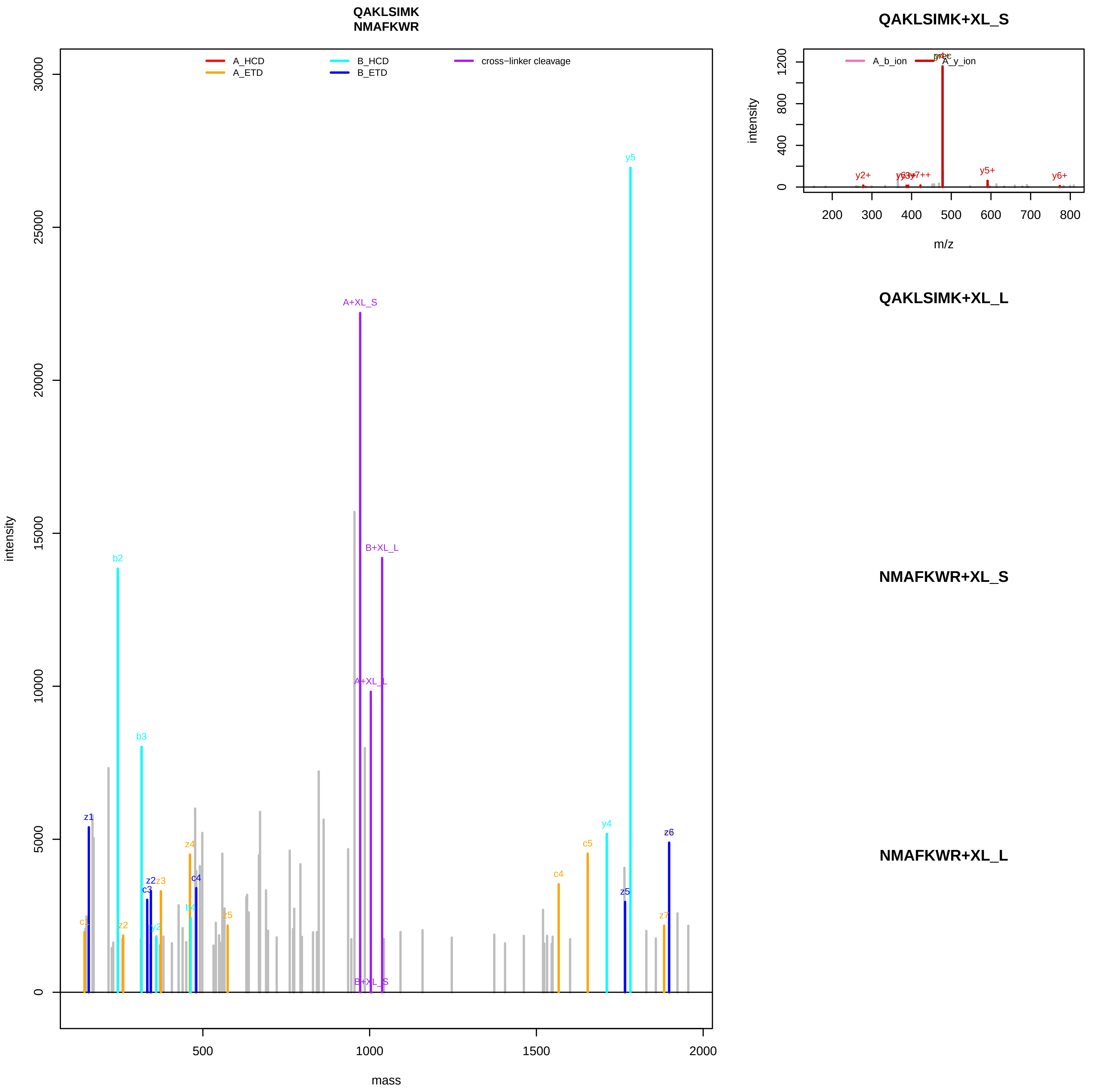
QAKLSIMK
NMAFKWR
A_HCD
A_ETD
B_HCD
B_ETD
cross−linker cleavage
intensity
mass
y5
A+XL_S
B+XL_L
A+XL_L
B+XL_S
b2
b3
b4
y2
y4
z1
z2
z3
z4
z5
z6
z7
c1
c3
c4
c5
QAKLSIMK+XL_S
A_b_ion
A_y_ion
y2+
y5+
y6+
m/z
QAKLSIMK+XL_L
NMAFKWR+XL_S
NMAFKWR+XL_L
